# Data on using macro invertebrates to investigate the biological integrity of permanent streams located in a semi-arid region

**DOI:** 10.1016/j.dib.2018.04.134

**Published:** 2018-05-05

**Authors:** Mostafa Karimaei, Mohammad Maroosi, Mansour Baziar, Hamed Biglari, Hooshmand Sharafi, Nezam Mirzaei, Amir Hossein Mahvi

**Affiliations:** a Department of Environmental Health Engineering, School of Public Health, Semnan University of Medical Sciences, Semnan, Iran; b Department of Environmental Health Engineering, School of Public Health, Neyshabur University of Medical Sciences, Neyshabur, Iran; c Department of Environmental Health Engineering, School of Public Health, Tehran University of Medical Sciences, Tehran, Iran; d Department of Environmental Health Engineering, School of Public Health, Gonabad University of Medical Sciences, Gonabad, Iran; e Students Research Committee, Kermanshah University of Medical Sciences, Kermanshah, Iran; f Department of Environmental Health Engineering, Faculty of Health, Kashan University of Medical Sciences, Kashan, Iran; g Center for Solid Waste Research, Institute for Environmental Research, Tehran University of Medical Sciences, Tehran, Iran; h National Institute of Health Research, Tehran University of Medical Sciences, Tehran, Iran

**Keywords:** Biological integrity, Tehran, Macro invertebrates

## Abstract

The aquatic ecosystems are continuously endangered due to variety of hazardous chemicals containing different toxic agents which can be emitted from anthropogenic sources. Besides the increasing of human population, various kinds of contaminants enter into the surface water resources. The aim of the present study was to investigate the abundance and diversity of macro invertebrates in two permanent streams located in the northern part of Tehran. The biological integrity of the streams was determined by manual sampling approach at five points. The distances between the sampling points were at least 2 km. The bio indicator organisms, organic pollution, and dissolved oxygen were measured. The different types of benthic invertebrates such as riffle beetle, midge and caddish fly larvae, dragon fly, may fly and stone fly nymph, riffle beetle adult, pyralid caterpillar, leech, and pouch snail were identified. It can be concluded that, the identified benthic macro invertebrates can be served as appropriate biological indicator in the studied area.

**Specifications Table**

**Table:** 

Subject area	Environmental Sciences
More specific subject area	Describe narrower subject area
Type of data	Tables and figures
How data was acquired	In this study, the sampling was gathered manually in five points with the spatial distance of two kilometer. In each point both bio indicators organism and organic pollution concentration and dissolved oxygen had been assessed. The different types of benthic invertebrates such as *Riffle Beetle*, *Midge* and *Caddish Fly Larvae*, *Dragon Fly*, *May Fly* and *Stone Fly Nymph*, riffle beetle adult, *Pyralid Caterpillar*, *Leech*, and *Pouch Snail* were identified. Identifying the collected macro invertebrates was achieved using the handy magnifier to observe the objects and an aquatic organism identification key. After identifying all organisms captured in the sampling tray, the trapped macro invertebrates into were returned into the aquatic ecosystem.
Data format	Raw, analyzed
Experimental factors	Identifying the collected macro invertebrates was achieved using the handy magnifier to observe the objects and an aquatic organism identification key.
Experimental features	The organic pollution concentration and dissolved oxygen, were analyzed according to the standards for water and wastewater treatment handbook.
Data source location	Darband and Darake area, Tehran, Tehran province, Iran
Data accessibility	Data are included in this article

**Value of the data**

• Quality survey of Darband and Darake rivers in term of organic pollutants by determination of Chemical oxygen demand (COD) and dissolved oxygen (DO). The quality of these two rivers is very important, because they are important in terms of tourism.
• Due to limited studies in the study area, the data of this study can help to better understand the organic and biological quality of rivers in the in northern part of Tehran and provide further studies.

## 1 Data

**Fig. 1:**
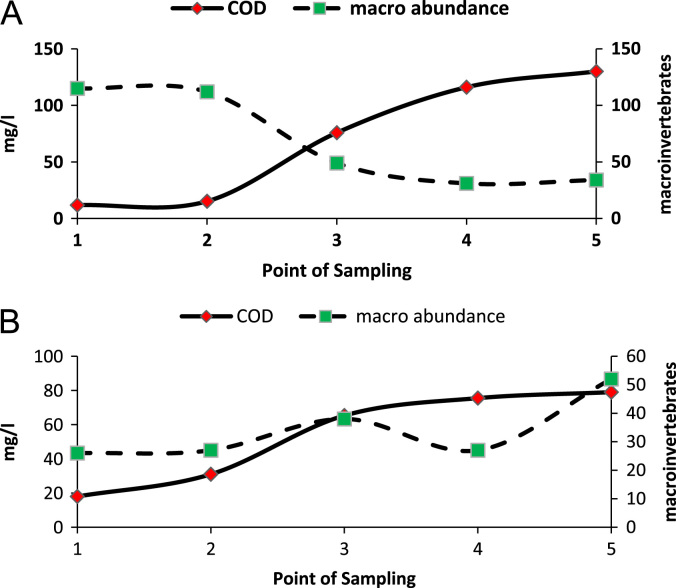
Trends of organic pollution concentrations and macro invertebrate׳s abundance along stream path Darband (A) and Darake (B).

Fig. 1 indicates that as pollution concentration levels reduced, benthic invertebrate abundance increased and there is inverse relationship between the number of organic pollution and those of benthic invertebrates.

**Fig. 2:**
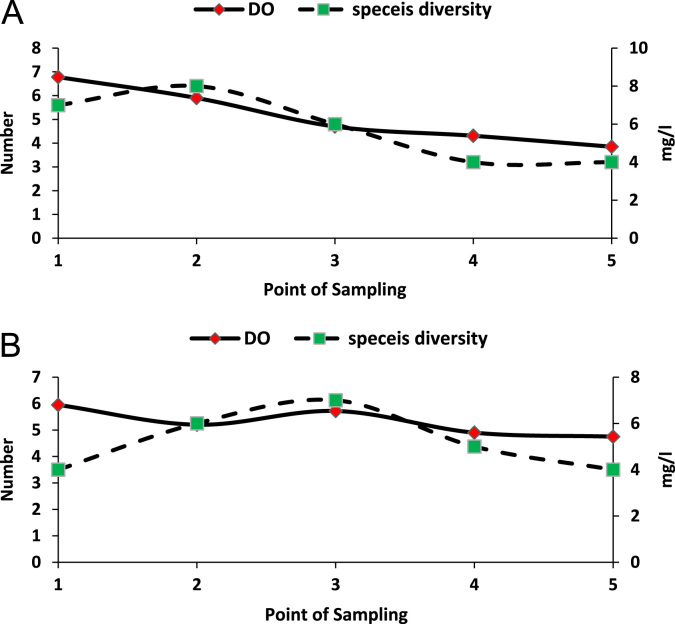
Spices diversity and dissolved oxygen variations across sampling location distance from urban area boundary. Darband streams (A) and Drake streams (B).

In addition, to COD and macro invertebrate abundance, dissolved oxygen of streams water and macro invertebrate has been assessed and compared in each sampling point which comparison of these two parameters demonstrated in Fig. 2. Altogether 9 and 7 different separate spices collected and found respectively in Darband and Darake riverbank, that variation of these spices in each point of sampling was also different. As shown in Fig. 2 by sampling from spring supplying streams water toward congested urban margins, number of traditional restaurants and recreational places gradually cut down, so dissolved oxygen of water in higher elevation sampling station increases remarkably. Diversity of macro invertebrate spices was also increased at the initial and then was decreased somehow during improvement the quality of streams water.

Based on the results provided in Table 1, different types of benthic invertebrates like *Riffle Beetle, Midge* and *Caddish Fly Larvae, Dragonfly, May Fly* and *Stone Fly Nymph, Riffle Beetle* Adult*, Pyralid Caterpillar, Leech* and *Pouch Snail* were identified on these streams. For example, *Riffle Beetle* and *Midge* and *Caddish Fly* Larvae, *Dragon Fly*, *May Fly* and *Stone Fly Nymph*, *Pyralid Caterpillar* and *Leech* and *Pouch Snail* collected and separated from different point of Darband streams and *Riffle Beetle* and *Caddish Fly* Larvae, *May Fly* and *Stone Fly Nymph*, *Riffle Beetle* Adult, *Leech* and *Pouch Snail* collected from Darake stream. According to Table 1 biological diversity in Darband water basin was more than Darake water basin.

**Table 1:** Different type of spices found in different parts of streams.

**Point of sampling**	**Riffle beetle larvae**	**Midge larvae**	**Caddish fly larvae**	**Leech**	**Pouch snail**	**May fly nymph**	**Stone fly nymph**	**Dragon fly larvae**	**Pyralide caterpillar**	**Riffle beetle adult**
**Spices collected and identified on Darband streams**										
Point 1	*		*		*	*	*	*	*	
Point 2	*		*	*	*	*	*	*	*	
Point 3	*		*	*	*	*	*			
Point 4	*		*	*	*					
Point 5	*	*		*	*					
**Spices collected and identified on Darake streams**										
Point 1	*			*	*	*	*			
Point 2	*			*	*	*	*			*
Point 3	*		*	*	*	*	*			*
Point 4	*			*	*	*	*			

In each part of the streams, the native species of macro invertebrates were detected. For instance, in the parts of streams located near the urban areas, which was more polluted, *Leeches and Pouch Snails* were the predominant spices and in the sampling point far from the urban areas which was relatively cleaner than other parts of streams spices such as *Stonefly* and *May Fly Nymph* and *Pyralid Caterpillar* were observed. In the middle parts laid between these extremes, the predominant spices were *Riffle Beetles* and the *Caddish Fly* larvae (Table 2). In addition classification of water quality based on COD and DO (Adopted from WHO/UNEP) presented in Table 3 [1].

**Table 2:** Predominant spices in different parts of streams.

**Point of sampling**	**predominant spices in Darband streams**	**Total number of macro invertebrate**	**predominant spices in Darake streams**	**Total number of macro invertebrate**
Point 1	pyralid caterpillar, May fly and stone fly nymph	115	Stone fly and may fly nymph	52
Point 2	Stone fly and may nymph	112	Riffle beetle larvae and may fly nymph	27
Point 3	Leach and riffle beetle larvae	49	Riffle beetle larvae and pouch snail	38
Point 4	Leech and caddish fly larvae	31	Riffle beetle larvae and stone fly nymph	27
Point 5	Leech and pouch snail	34	Leech and pouch snail	26

**Table 3:** Classification of water quality based on COD and DO [1].

**Variables**	**Class 1**	**Class 2**	**Class 3**	**Class 4**	**Class 5**
COD mg/l	<3	3–10	10–20	20–30	30<
DO mg/l	7<	6–7	4–6	3–4	<3

Adopted from WHO/UNEP (Helmer et al., 1997).

## 2 Experimental design, materials and methods

### 2.1 Description of study area

**Fig. 3:**
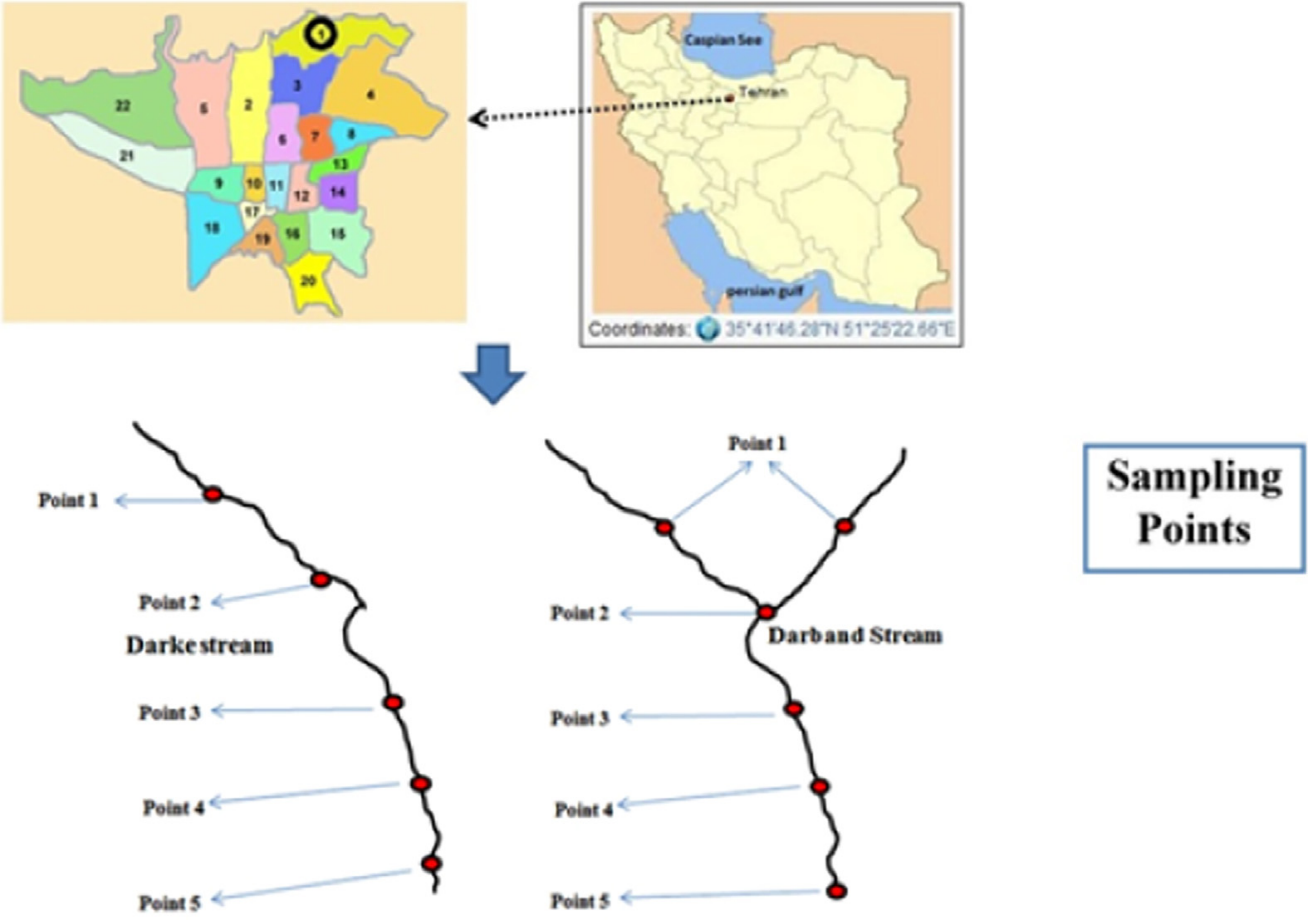
Map of sampling points.

This study was carried out on two permanent streams in the mountainous area in the northern part of Tehran metropolitan called Darband and Darake. These streams are important for tourism purposes. Fig. 3 shows the sampling points and the study region in the Alborz Mountains.

Based on the precipitation data received from meteorology organization, the annual precipitation is 900–1000 mm/year. The areas located in northern part of Tehran were mainly covered by Mediterranean vegetation.

The aim of this descriptive study is to investigate the abundance and variety of macro invertebrate and ultimately to determine the biological integrity condition of these streams.

Unit of the hydrological basin of Darband streams include of two sub basins: one of them is Jafarabad with area of 865 ha and the other one is Osoon hydrological catchment with the area of 978 ha. Both of these streams originated from Tochal peak and join together and form the Darband streams and flows in Darband resort canyon then continued to the urban area of tajrish in Tehran. Average flow rate of Darband stream is 0.42 m^3^/s. Notable point on the path of Darband stream is existence of gardens along the edge of river upstream and numerous traditional Restaurant and resorts along the edge of river downstream until the Sarband square area, which is increase the likely of stream pollution to the variety of natural and synthetic pollutants. It should be noted that flow rate of this river is especially significant in spring season, So that the river floods occurred in 1987 and destroyed the Tajrish market in downstream urban area.

Hydrological basin of Darake steam covers an area of 2200 ha with the highest branch of the 9.7 km long, which originated from a height of 3550 m and then continued till the Evin area in northern part of Tehran city and after crossing the Evin-Saadat Abad Bridge enters the ravine beside of chamran highway stream. According to12-year statistics of Darake streams, the average flow rate of this stream is around 0.56 m^3^/s. Margins of this river in upstream seem to be subjected to the agricultural activities and in part of water basin located near the urban area, pollution from the resorts and restaurants around river bed appear affect the biological quality of Darake stream [2].

### 2.2 Method and material

In this study, the sampling was gathered manually in five points with the spatial distance of two kilometer. In each point both bio indicators organism and organic pollution concentration and dissolved oxygen had been assessed. Sampling begins from the source of streams goes to the entry point of them to urban area. Had been planned to sample in three different spots within a 100-yard stream reach. Pick up any large rocks in the 3-foot by 3-foot sampling area and rub them thoroughly over the partially-filled bucket so that any macro invertebrates clinging to the rocks will be dislodged into the bucket. Also net was used for collecting macro invertebrate in each 9foot^2^ (3*3) sampling area and working downstream and removing macro invertebrates from the net on the water bucket. Any large rocks used to anchor the net should be thoroughly rubbed into the bucket as above. Identifying the collected macro invertebrates was achieved using the handy magnifier to observe the objects and an aquatic organism identification key. After identifying all organisms captured in the sampling tray, the trapped macro invertebrates into were returned into the aquatic ecosystem. If identifying a certain organism was not feasible, one or two specimens were sent to the laboratory under the preservation in alcoholic solution. On your field data sheet, note the number of individuals of each type of organism you have identified. All sampling and experimental methods were performed according to the standard methods [3,4].
