# Supplementary material for: Data on using macro invertebrates to investigate the biological integrity of permanent streams located in a semi-arid region
Source: Data Brief. 2018 May 5;19:542–7. doi: 10.1016/j.dib.2018.04.134 (PMC5997610; doi:10.1016/j.dib.2018.04.134)
Supplement: Supplementary file 1 — Supplementary material [file mmc1.docx]

**Compliance with Ethical Standards**

• Authors are aware of, and comply with, best practice in publication ethics specifically with regard to authorship (avoidance of guest authorship), dual submission, and manipulation of figures, competing interests and compliance with policies on research ethics.

• All authors have consented, read and been familiar with this study.

• Authors confirm that research does not involve Human participants or Animals.

• Authors adhere to publication requirements that submitted work is original and has not been published elsewhere in any language.

• All authors present results of the study honestly and without fabrication, falsification or inappropriate data manipulation.

• Authors take collective responsibility for submitted and published work.

• The authors declare no conflict of interest.

Sincerely yours,

Dr. Amir Hossein Mahvi
